# A Novel Series Connected Batteries State of High Voltage Safety Monitor System for Electric Vehicle Application

**DOI:** 10.1155/2013/261313

**Published:** 2013-09-30

**Authors:** Qiang Jiaxi, Yang Lin, He Jianhui, Zhou Qisheng

**Affiliations:** School of Mechanical Engineering, Shanghai Jiaotong University, Shanghai 200240, China

## Abstract

Batteries, as the main or assistant power source of EV (Electric Vehicle), are usually connected in series with high voltage to improve the drivability and energy efficiency. Today, more and more batteries are connected in series with high voltage, if there is any fault in high voltage system (HVS), the consequence is serious and dangerous. Therefore, it is necessary to monitor the electric parameters of HVS to ensure the high voltage safety and protect personal safety. In this study, a high voltage safety monitor system is developed to solve this critical issue. Four key electric parameters including precharge, contact resistance, insulation resistance, and remaining capacity are monitored and analyzed based on the equivalent models presented in this study. The high voltage safety controller which integrates the equivalent models and control strategy is developed. By the help of hardware-in-loop system, the equivalent models integrated in the high voltage safety controller are validated, and the online electric parameters monitor strategy is analyzed and discussed. The test results indicate that the high voltage safety monitor system designed in this paper is suitable for EV application.

## 1. Introduction

Over the last several years, air pollution and energy shortage have led to a number of initiatives to develop EV which usually includes PEV (Pure Electric Vehicle), HEV (Hybrid Electric Vehicle), and FCEV (Fuel Cell Electric Vehicle). Batteries, as the main or assistant power source of EV, are usually connected in series with high voltage to improve the drivability and energy efficiency. The high voltage of series connected batteries in some applications almost reaches to 600 V [1] which is high enough to bring deadly danger to people. Moreover, the current of high voltage bus is usually up to hundreds of Amperes when the vehicle is operating regularly. If there is any fault in HVS, the consequence is serious and dangerous. Thus, the corresponding high voltage standards are presented and implemented. Based on the precondition that the maximal voltage is lower than 660 V (AC) and 1000 V (DC), the high voltage standards of EV are summarized as following [2–4].

- It should need 100 milliseconds at least to close relays. The precharge process should be adopted to avoid the high voltage impulse when the ignition key is turned on.
- The insulation resistance that divides the voltage of battery pack should be greater than 100 Ω/V at least, and it is better to ensure that it is greater than 500 Ω/V.
- The disconnection time of relay must be less than 20 milliseconds. The peak voltage between any electric part of vehicle and ground should be lower than 42.4 V (AC) and 60 V (DC), and the residual energy should be less than 20 J after relays are cut off for one second.

It is necessary to develop a special system which can monitor the electric parameters of HVS and implement the corresponding control strategy real-time to ensure the safety of people and vehicle. Although the high voltage safety management for series connected batteries is very important, only a few studies of it can be found. In this paper, a high voltage safety management system is summarized as four equivalent models including precharge model, HVIL (High Voltage Inter Lock) model, insulation resistance model and remaining capacity model, and analyzed by the hardware-in-loop simulation. The high voltage safety controller based on the analysis in this paper has been successfully used in the QRPEV (CHERY Pure Electric Vehicle) [5].

## 2. High Voltage System Configuration of EV

**Figure 1:**
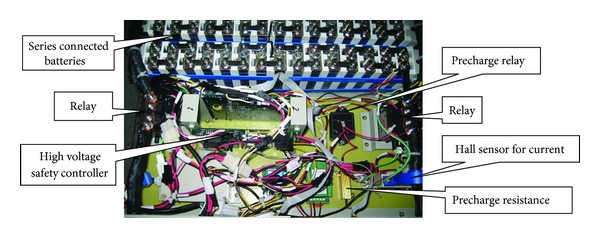
The related components of high voltage safety management system.

Some particular means are adopted to insure the high voltage safety of EV. Firstly, the series connected batteries and related high voltage components are packed in a locked and insulated box, and the relays are installed near the terminals of battery to cut off the output of high voltage when there is any fault in HVS. Secondly, the high voltage safety controller should also be installed in the box to monitor high voltage parameters and share the information with the central controller PTCM (Power Train Control Module). The related components of high voltage management system are shown in Figure 1 [6, 7].

**Figure 2:**
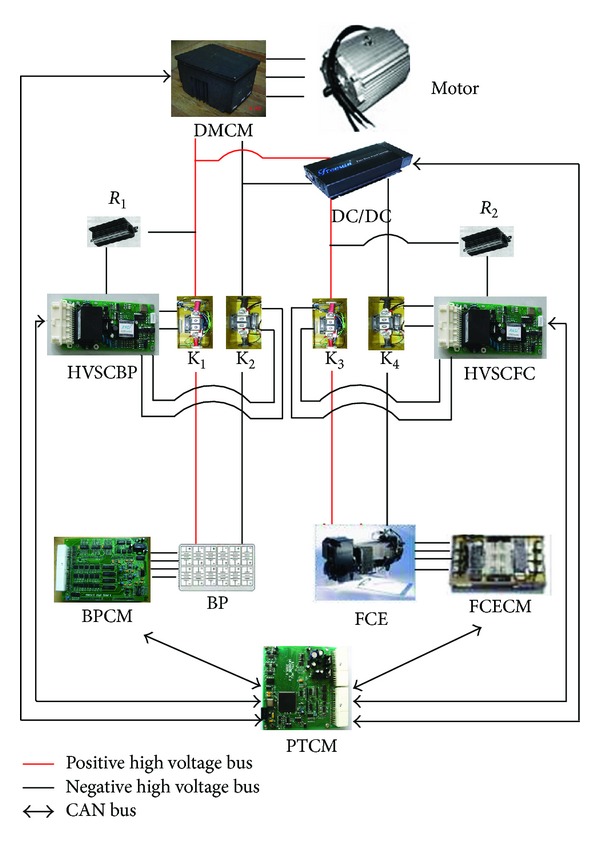
The high voltage system configuration of FCEV.

The high voltage system configuration of FCEV is shown in Figure 2 for instance. There are two power sources BP (Battery Pack) and FCE (Fuel Cell Engine) in FCEV. K_1_ and K_2_ are controlled by HVSCBP (High Voltage Safety Controller for Battery Pack) to manage the output of high voltage of battery pack. K_3_ and K_4_ are controlled by HVSCFC (High Voltage Safety Controller for Fuel Cell) to manage the output of high voltage of fuel cell. *R*_1_ and *R*_2_ are precharge resistances for battery pack and fuel cell, respectively. The central controller of FCEV is PTCM. The other controllers share information with PTCM by CAN (Controller Area Network). The PTCM will send the command to HVSCBP and HVSCFC according to the integrated information of vehicle [8].

## 3. High Voltage Safety Management System Structure 

**Figure 3:**
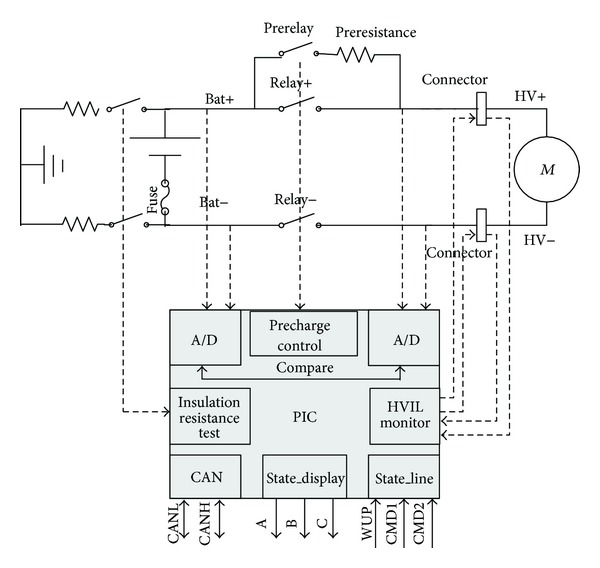
The structure of the high voltage safety management system.

In this study, the high voltage safety management system is based on PIC Microchip which makes it easily to share information among controllers by CAN and can be operated by the flexible program technology.  Figure 3 shows the structure of the high voltage safety management system. The system is developed to solve the critical issues including precharge control, HVIL monitor, insulation resistance monitor, and remaining capacity monitor. Three relays (Prerelay, Relay+, and Relay−) control the precharge process and Relay+ and Relay− manage the high voltage output of series connected batteries. The high voltage impulse can be avoided when the three relays are controlled by the logical control order. HVIL checks the connection status of high voltage bus by measuring the contact resistance of high voltage bus. The high voltage safety management system monitors the insulation resistance by the known resistance which is inserted between the terminals of battery and ground. In order to monitor remaining capacity, an equivalent circuit model is established to calculate the residual energy. Two communication modes are adopted to improve the system reliability: one is CAN which has the higher priority, and the other is state line. The high voltage safety management system is the integration of electric parameters monitor and faults diagnosis. The diagnosis code is displayed by the combination of A, B, and C shown in Figure 3. 

## 4. Equivalent Circuit Models

### 4.1. Precharge Model

**Figure 4:**
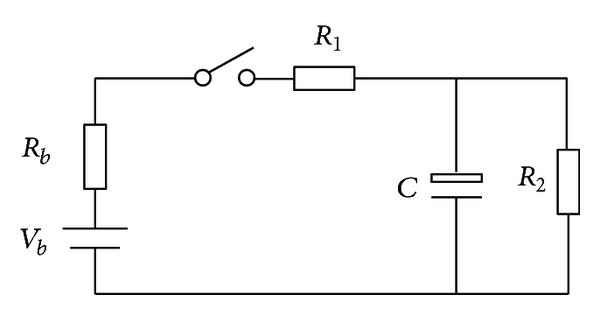
The precharge model.

**Figure 5:**
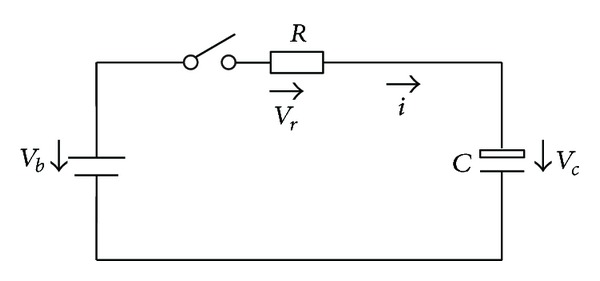
The simplified precharge model.

If the relays (Relay+ and Relay−) are switched on directly, the high voltage will impulse the electric system for the input capacitance of high voltage equipment. Moreover, if there is any fault with HVS, it will cause the dangerous accident. The precharge process can solve these problems. The specific method is that before each start process, Prerelay and Relay− (shown in Figure 3) are closed first. If the voltage between HV+ and HV− reaches certain value (90% of BP's voltage defined in this study) in time, Relay+ and Relay− are permitted to be closed, and Prerelay is cut off, or all relays are cut off and output the precharge fault. The precharge model shown in Figure 4 is proposed through analyzing the HVS configuration and precharge curves. In Figure 4, *V*_*b*_ is the voltage of battery pack, *R*_*b*_ is the battery pack internal resistance, *R*_1_ is precharge resistance, *R*_2_ is equivalent resistance of HVS, and *C* is the equivalent capacitance of HVS. Among these parameters, *V*_*b*_ can be measured, and *R*_*b*_ is very small. During the precharge process, all equipments are forbidden to work which means that *R*_2_ is very large. In order to simplify the computing, *R*_*b*_ and *R*_2_ are ignored according to the above discussion. The precharge model shown in Figure 4 is simplified as Figure 5 shows.

According to Kirchhoff's law, there are

(1) Vb=Vr+Vc,Vr=R∗i,i=C∗dVcdt.

By the combination of (1), there is

(2) R∗C∗dVcdt+Vc=Vb.

Considering the initial condition that *t* = 0, *V*_*c*_ = 0

(3) Vc=Vb(1−e−t/RC).

So the equivalent capacitance can be calculated as:

(4) C=−t(R∗ln⁡(1−Vc/Vb)).

According to the actual precharge process, the equivalent capacitance of HVS can be calculated according to the known precharge resistance and precharge time (*t*). Then the precharge resistance can be selected based on the calculated equivalent capacitance and actual situation to minimize the precharge time. 

### 4.2. HVIL Model [9]

**Figure 6:**
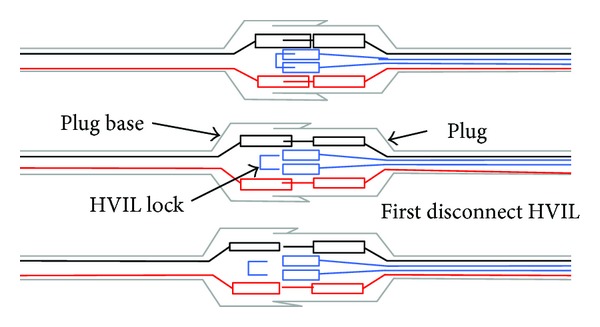
High voltage connector used for EV.

HVIL checks the connection status of high voltage bus. Two parallel methods are used in this work to improve the system reliability. The position where is easy to lose connection such as connectors shown in Figure 6 uses low voltage circuit loop to measure the accurate contact resistance. As shown in Figure 6, the high voltage connector integrates the HVIL in the plug, and when the connector is loose, the HVIL is disconnected first to inform the high voltage management system. 

**Figure 7:**
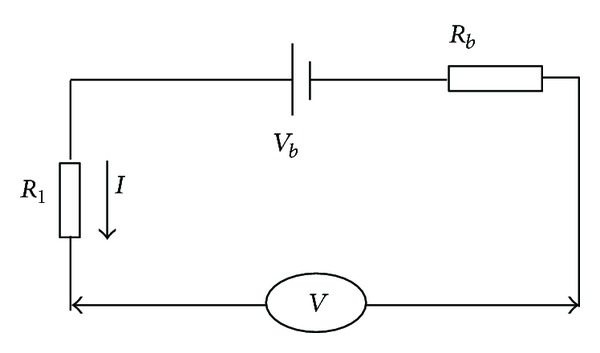
The HVIL model.

However, not all the connected points in the HVS have the same structure shown in Figure 6, and the overall contact resistance is calculated according to the variable voltage and current to monitor the connection state of HVS. The equivalent HVIL calculation model is shown in Figure 7. In Figure 7, the battery model is simplified as the series connection of unit voltage source *V*_*b*_, battery pack internal resistance *R*_*b*_, line resistance *R*_1_, and the voltage of HVS (*V*).

According to Kirchhoff's law, there is

(5) Rb+R1=(Vb−V)I.

Assuming that the voltage of HVS is *V*_11_ and current is *I*_1_ in time step *t*_1_ and the voltage of HVS is *V*_22_ and current is *I*_2_ in time step *t*_2_, there are

(6) Rb+R1=(Vb−V11)I1,Rb+R1=(Vb−V22)I2.

By the combination of (5), (6), there is:

(7) Rb+R1=(V22−V11)(I1−I2).

**Figure 8:**
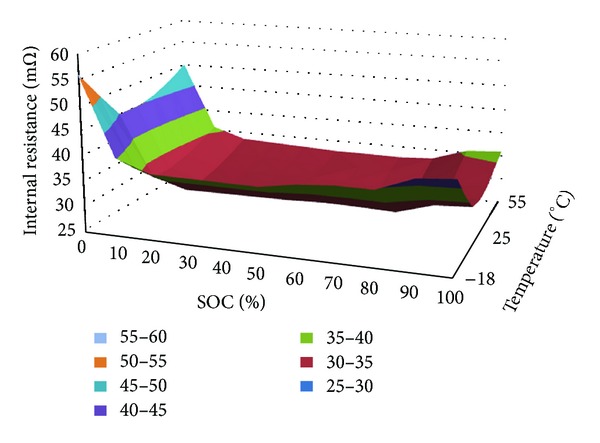
Battery internal resistance under different SOC and temperature.

The line connection status can be checked by the formula (7). The fault level of connection status is differentiated according to the different contact resistance. The drawback of (7) is that the internal resistance *R*_*b*_ of battery pack is variable when the ambient temperature and SOC (State of Charge) are different. Therefore, the prudent method adopted in this study is that the *R*_*b*_ is interpolated form the table which is made up of *R*_*b*_, temperature, and SOC recorded in the laboratory.  Figure 8 shows the NiMH battery module internal resistance in different temperature and SOC [10]. Then it is reasonable to trust that *R*_1_ calculated from (7) has the high accuracy if voltage and current are measured accurately. 

### 4.3. Insulation Resistance Model [11]

**Figure 9:**
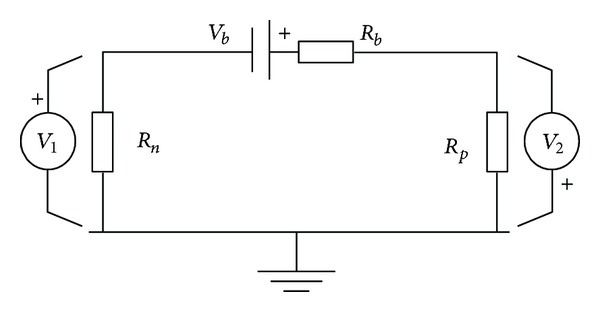
The insulation resistance model.

The insulation resistance is the key parameter to evaluate the high voltage safety state. If the insulation level is low, passengers may get electric shock. According to the standard, the insulation resistance must be greater than 100 Ω/V. The insulation resistance calculation model is shown in Figure 9. *R*_*p*_ is positive insulation resistance, and *R*_*n*_ is negative insulation resistance. *V*_*b*_ is the voltage of battery pack, and *R*_*b*_ is battery pack internal resistance which can be ignored compared to *R*_*n*_ and *R*_*p*_. 

According to Kirchhoff's law, there are

(8) V1+V2=Vb,V1Rn=V2Rp.

**Figure 10:**
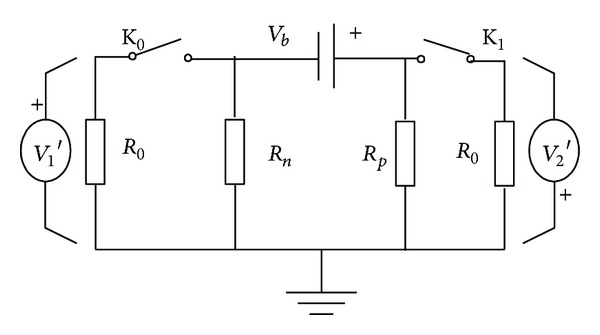
Calculation model of positive insulation resistance.

*R*_0_ is a known resistance which is inserted between battery terminal and ground in Figure 10. The interference of *R*_0_ should not change the insulation level of HVS. When the switch K_0_ is closed and the K_1_ is open, according to Kirchhoff's law, there are

(9) V1′+V2′=Vb,V1′R0+V1′Rn=V2′Rp.

According to (8)-(9) and the known *R*_0_, the formula which can calculate the positive insulation resistance is defined as

(10) Rp=R0(V2′V1′−V2V1).

The negative insulation resistance is defined as

(11) Rn=R0(V2′∗V1V2∗V1′−1).

Also, the positive and negative resistances can be obtained in the following states:

1. K_0_ and K_1_ are open, K_0_ is open and K_1_ is closed;
2. K_0_ is open and K_1_ is closed, K_0_ is closed and K_1_ is open.

**Figure 11:**
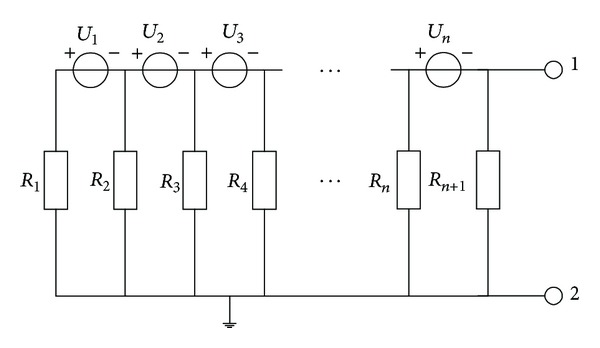
The internal insulation resistance of series connected batteries.

**Figure 12:**
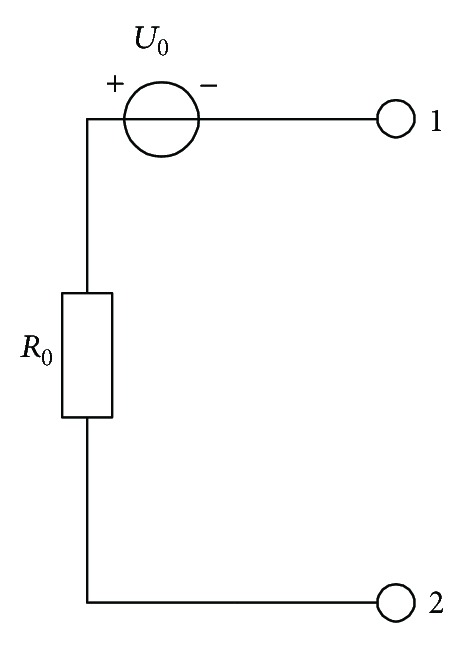
Simplified insulation model.

The above discussion solves the insulation problem of battery positive and negative terminal, but the weak insulation points usually exist in the middle of the series connected batteries, as shown in Figure 11. In this situation, the equivalent parallel insulation resistance is calculated. The model shown in Figure 12 can be simplified as a constant voltage source and a series connected resistance as Figure 11 shows. 

According to Kirchhoff's law, there are

(12) U0=(U1R1+U2R12+U3R123+⋯+UnR123…n)×R123…n(n+1),R0=R123…n(n+1),

where *R*_123…*n*(*n*+1)_ is the parallel value of *R*_1_, *R*_2_, *R*_3_ … *R*_*n*_.

The *R*_0_ is the system parallel insulation resistance, and it can be calculated by inserting different resistances (*R*_*n*1_, *R*_*n*2_) between terminals 1 and 2 shown in Figure 11. According to Kirchhoff's law, there are

(13) Vn1rev=Rn1R0+Rn1×U0,Vn2rev=Rn2R0+Rn2×U0.

According to (13), the equivalent system parallel resistance *R*_0_ is defined as

(14) R0=(Vn2rev−Vn1rev)×Rn1×Rn2Vn1rev×Rn2−Vn2rev×Rn1.

Also the weak insulation position can be fixed by calculating the insulation voltage defined in

(15) U0=Vn1revRn1×(R0+Rn1).

### 4.4. Remaining Capacity Model

**Figure 13:**
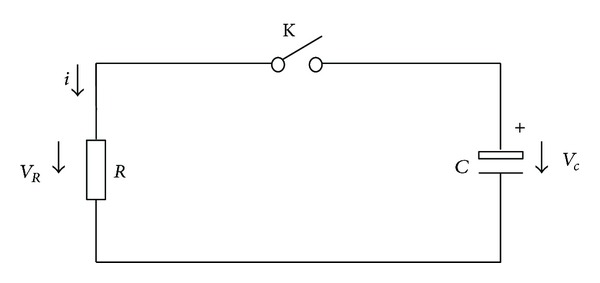
The remaining capacity model.

When the relays are cut off, the remaining capacity mainly concentrates on the equivalent capacitance of the high voltage bus.  Figure 13 shows the remaining capacity model. *V*_*c*_ is the voltage of the equivalent capacitance of high voltage bus, *V*_*R*_ is the voltage of the equivalent resistance of high voltage bus, and *i* is the release current. 

According to Kirchhoff's law, there are

(16) VC=VR,VR=R∗i,i=−C∗dVCdt.

In the initial state, *t* = 0, *V*_*c*_ = *V*_0_, and *V*_0_ is equal to the voltage of battery pack before the relays are cut off.

The capacity release time can be calculated by the combination of formulas (16):

(17) t=CR(ln⁡V0−ln⁡Vt).

The equivalent capacitance *C* of HVS can be calculated by the formula (4). Assuming that the voltage of high voltage bus is *V*_*t*_ at time step *t* after relays are cut off, the residual energy *W* can be calculated as

(18) W=C∗Vt22.

## 5. Experiment Validation and Discussion [12]

**Figure 14:**
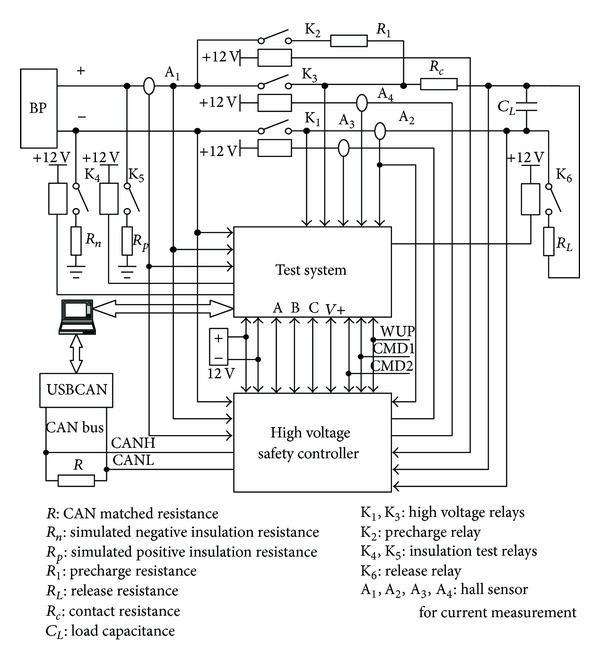
Circuit structure schematic diagram of high voltage safety test.

**Figure 15:**
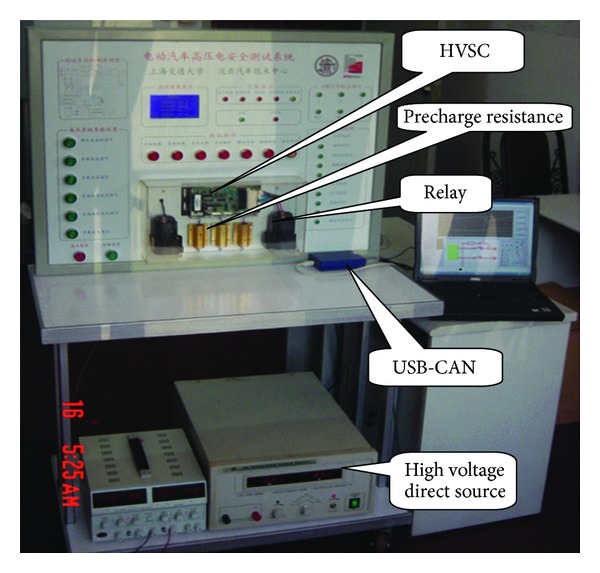
The actual experiment table.

A hardware-in-loop system shown in Figure 14 is developed to validate the proposed equivalent circuit models and study on the control strategy.  Figure 15 is the actual test table. Signals in the hardware-in-loop system are listed and explained as follows.

*Nine Analog Input Signals*. Five voltage signals are the voltage between battery positive terminal and ground, the voltage between battery negative terminal and ground, the output voltage between positive high voltage bus and ground, and the output voltage between negative high voltage bus and ground, the operating voltage of high voltage safety controller (V+).

Four current signals are the current of positive high voltage bus, the current of negative high voltage bus, the coil current of positive relay, the coil current of negative relay.

*Six Input Switch Signals*. Three high voltage safety controller commands to manage the output of high voltage: WUP, CMD1, and CMD2.

Three high voltage system states to display the diagnosis code: A, B, and C.

*Six Output Switch Signals*. K_1_, K_2_, K_3_, K_4_, K_5_, and K_6_.

### 5.1. Experiment and Analysis for Precharge Model

**Figure 16:**
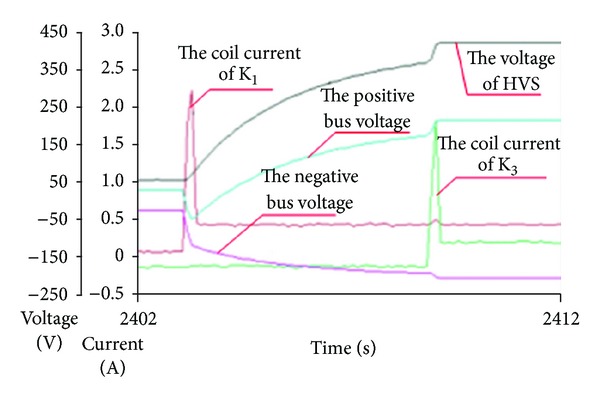
The test trial curve of high voltage precharge.

Figure 16 shows the precharge curves. The test condition is as follows the voltage of battery pack is 420 V, the precharge resistance is 8364 Ω, and the equivalent capacitance is 330 uF. The recorded precharge time is 6.5 seconds, and the theoretical value calculated by the precharge model is 6.36 seconds. The fact indicates the accuracy of the precharge model. In Figure 16, the coil current of relay is also monitored because it can indicate the working state of relay. Two peak current pulses indicate the close sequence of relays during precharge process. 

**Figure 17:**
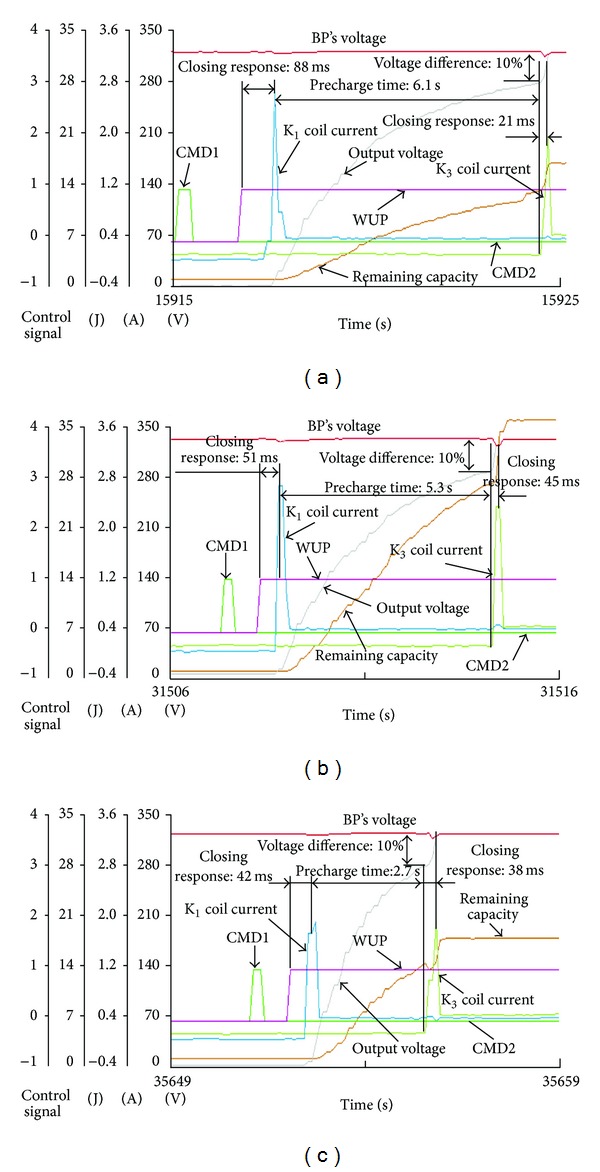
The contrastive experiment of different precharge resistance and load capacitance.

The contrastive test results are shown in Figure 17 to analyze the control strategy for precharge. The voltage of battery pack is 326 V in the contrastive test. The precharge resistance is 8364 Ω, and the load capacitance is 330 uF in Figure 17(a), 3637 Ω and 660 uF in Figure 17(b), and 3637 Ω and 330 uF in Figure 17(c). During the test circulation, the precharge time of each test condition calculated by the equivalent model is 6.1 s, 5.3 s, and 2.7 s, respectively, and the calculation error of load capacitance is −3.6%, 1.8%, and 0.6%. Through analyzing the different test curves in Figures 17(a), 17(b), and 17(c), two visualized conclusions can be got:

- the shorter time precharge costs, the less error of load capacitance calculation,
- the less value of precharge resistance and load capacitance, the shorter time precharge costs.

It is valuable to select the proper precharge resistance according to the actual situation in order to reduce precharge time and avoid high voltage impulse. From Figure 15, the relays are controlled by the combination of commands WUP, CMD1 and CMD2. The relays are permitted to be closed only when WUP is logic “1,” and both CMD1, and CMD2 are logic “0.” This combined command method can avoid mishandling and interference. The logic relay control strategy of precharge process is as follows: the precharge and negative relays are closed firstly, then the positive relay is closed, and the precharge relay is cut off when the voltage of the high voltage bus reaches to the 90% of battery pack voltage in time to insure that there is no fault with HVS, or all the relays are forbidden to be closed and output the precharge fault. 

### 5.2. Experiment and Analysis for HVIL Model

**Figure 18:**
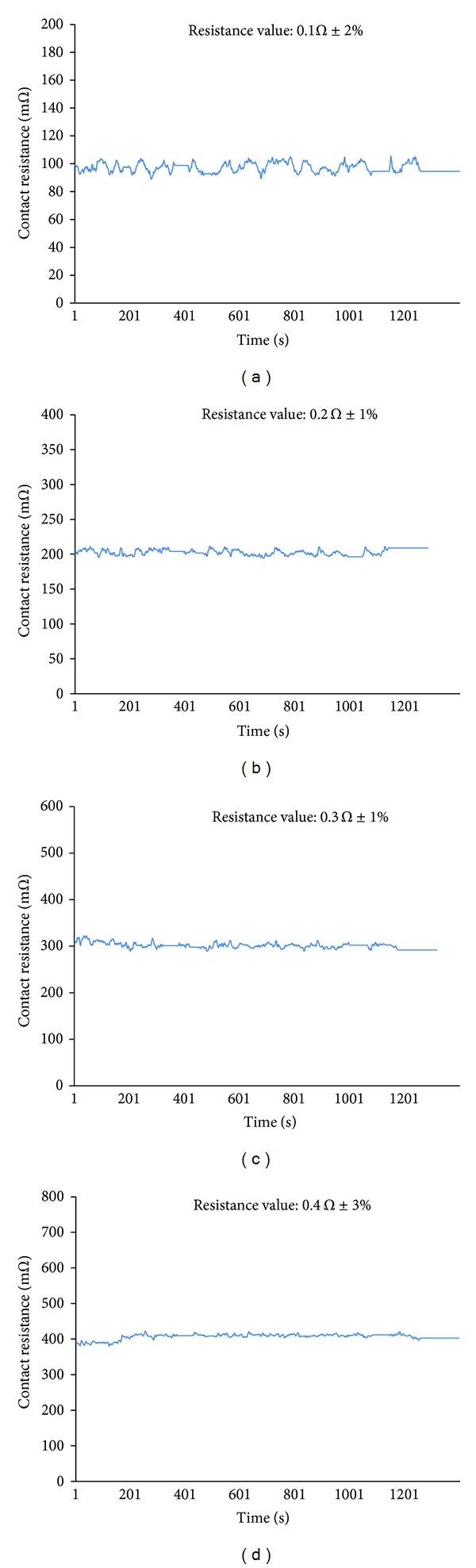
Contact resistance model and calculation algorithm verification.

The HVIL model discussed in Section 5 is the combination of battery internal resistance and line resistance monitor. It is necessary to consider the variable battery internal resistance influenced by SOC, temperature, and charge/discharge current. A wise method adopted in this study is that the whole HVS is tested in the laboratory under simulating the complicated drive cycle. The safe contact resistance range defined in this work is 0.1 Ω~1 Ω according to the experimental results. The contact resistance computed by formula (7) is based on the cautious choice of referenced current (*I*_1_, *I*_2_) and voltage (*V*_11_, *V*_22_). Four different contact resistances (0.1 Ω, 0.2 Ω, 0.3 Ω, and 0.4 Ω) are connected to the high voltage bus to verify the contact resistance model and calculation strategy discussed above. The test results are shown in Figure 18. The calculation error is between 1%~6% which indicates that its accuracy is high enough for the EV application. 

### 5.3. Experiment and Analysis for Insulation Resistance Model

It is not convenient to verify the insulation resistance model directly. Therefore, the known resistances (*R*_*n*_ and *R*_*p*_) are put into the test circuit to act as the negative and positive insulation resistances, respectively. The high voltage safety controller calculates positive and negative insulation resistances according to the equivalent circuit model. The accuracy of the equivalent circuit model can be verified via comparing the calculation result to the known value. The contrastive test results are shown in Table 1. From Table 1, the calculated insulation resistance is very close to the real value which validates the equivalent circuit model. 

**Figure 19:**
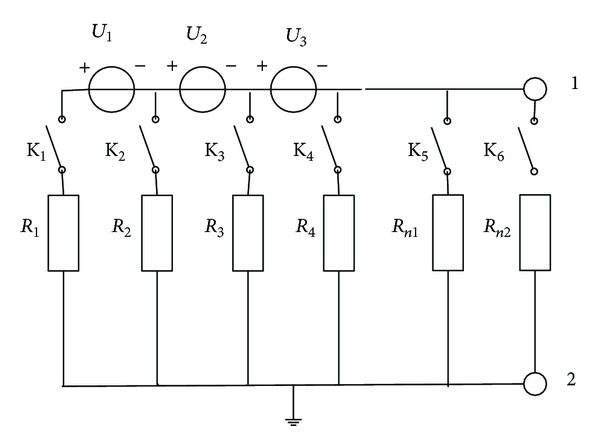
Simplified internal insulation resistance test model.

**Figure 20:**
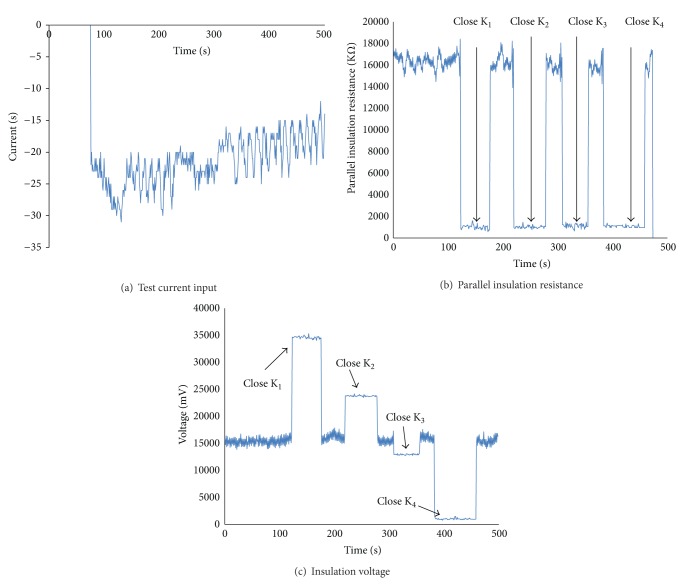
Parallel insulation resistance and insulation voltage test.

In order to verify the internal insulation resistance model, a simplified test model is set up as Figure 19 shows. Three lead-acid batteries which nominal voltage is 12 V are connected in series, and in each connection point, a known resistant is controlled by the switch (K_1_, K_2_,…, K_6_) whether it is connected to the ground. The resistance value is shown in Table 2, and the test results are shown in Figure 20.  Figure 20(a) shows the current input during the test cycle. Once one of the four switches (K_1_, K_2_, K_3_, and K_4_) closes, the parallel insulation resistance drops to the value which is near to the closed resistance. The insulation voltage is calculated by (15); when the switch K_1_ is closed, the insulation voltage is near to 35 V, and this indicates that the weak insulation position is near to the positive terminal of *U*_1_. Also, when the switch K_2_ is closed, the insulation voltage is near to 24 V, and this indicates that the weak insulation position is near to the positive terminal of *U*_2_. So the internal weak insulation position can be fixed by the calculated insulation voltage compared to the nominal voltage of battery cell.

### 5.4. Experiment and Analysis for Remaining Capacity Model

**Figure 21:**
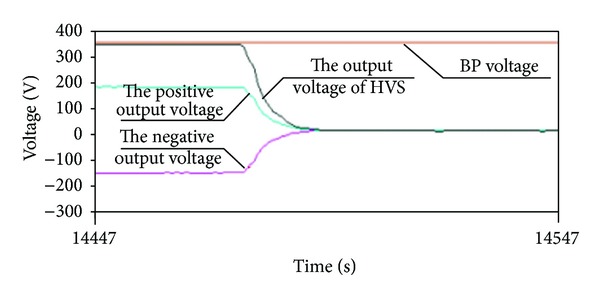
The test trial curve of high voltage discharges.

The remaining capacity of HVS when the relays are cut off is usually a neglected problem. People sometimes misjudge that the HVS is safe when the relays are cut off to stop the high voltage output of series connected batteries. However, there is much remaining electricity stored in the equivalent capacitance of high voltage bus which may hurt the human body. An experiment was done to validate the remaining capacity model. The test condition is as follows: the voltage of battery pack is 320 V, the equivalent discharge resistance (*R*_*L*_) is 200 Ω, and the equivalent capacitance of high voltage bus is 19800 uF. According to formulas (17) and (18), the theoretical duration of the voltage release to 60 V is 6.601 s. The actual duration (6.5 s) is close to the theoretical value which validates the equivalent model. The test results are shown in Table 3. In order to insure voltage and residual energy release to the safe level in time after relays are cut off, a wise method is that a small superpower discharge resistance is switched on to provide a quick path for voltage and residual energy release. Figure 19 shows the release curve of the equivalent capacitance of high voltage bus. The discharge resistance is selected to be 15 Ω to substitute primary 200 Ω. From Figure 21, the duration of the voltage release to 60 V is 0.5 second, and the calculated residual energy after the relays are cut off for 1 second is 1.206 J. It is meaningful to choose the proper discharge resistance according to the equivalent capacitance of high voltage bus to insure the remaining capacity safety.

## 6. Conclusions 

The high voltage safety management system proposed in this paper is the combination of electric parameters monitoring and fault diagnosis. Four equivalent circuit models including precharge model, HVIL model, insulation resistance model, and remaining capacity model are integrated in the high voltage safety management system for electric parameters monitor. A high voltage safety controller is developed and validated by the help of hard-ware-in-loop system. The high voltage electric parameters monitor strategy is presented based on the test results. The proposed high voltage management system in this paper has the instructional meaning for the safety design of EV. 

## Figures and Tables

**Table 1:** Static insulation resistance test result.

Positive high voltage bus	Inserted resistance (kΩ)	1000	500	250	100	50	2000	2000	2000	2000	2000
	Measured resistance (kΩ)	988	492	247	99	49	1960	1952	1958	1956	1950
	Error (%)	1.2	1.6	1.2	1.0	2.0	2.0	2.4	2.1	2.2	2.5

Negative high voltage bus	Inserted resistance (kΩ)	2000	2000	2000	2000	2000	1000	500	250	100	50
	Measured resistance (kΩ)	1948	1950	1946	1954	1944	984	491	247	98	49
	Error (%)	2.6	2.5	2.7	2.3	2.8	1.6	1.8	1.2	2.0	2.0

**Table 2:** Different insert resistance value.

	*R*_1_	*R*_2_	*R*_3_	*R*_4_	*R*_*n*1_	*R*_*n*2_
Value kΩ	0.986	0.986	0.988	0.990	19.84	56.69

**Table 3:** Test result of remaining capacity.

Test item	The test result of experiment										Average value	Theoretical value
	Times											
	1	2	3	4	5	6	7	8	9	10		
Discharge resistor (KΩ)	196	200	202	199	205	198	203	197	204	199	200.3	200
The equivalent capacitance (uF)	19250	20019	19905	20101	18965	19602	20284	19778	20041	19230	19717.5	19800
The dump energy (J)	34.65	36.03	35.83	36.18	34.14	35.28	36.51	35.60	36.07	34.61	35.49	35.64
Time of droping voltage(s)	6.445	6.702	6.664	6.730	6.349	6.563	6.791	6.622	6.710	6.438	6.601	6.629
